# Supplementary material for: Modeling the impact of COVID-19 on future tuberculosis burden
Source: Commun Med (Lond). 2022 Jun 29;2:77. doi: 10.1038/s43856-022-00145-0 (PMC9243113; doi:10.1038/s43856-022-00145-0)
Supplement: Supplementary file 1 — Supplementary materials [file 43856_2022_145_MOESM1_ESM.pdf]

Modeling the impact of COVID-19 on future tuberculosis burden
Supplementary Information

Mario Tovar,[1,2] Alberto Aleta,[3] Joaquín Sanz,[1,2] and Yamir Moreno[1,2,3]

[1] *Institute for Biocomputation and Physics of Complex Systems (BIFI), University of Zaragoza, Zaragoza 50009, Spain*
[2] *Department of Theoretical Physics, University of Zaragoza, Zaragoza 50009, Spain*
[3] *ISI Foundation, Via Chisola 5, 10126 Torino, Italy*

(Dated: June 15, 2022)

CONTENTS

Supplementary Methods 1

Supplementary Notes 1 6

Supplementary Notes 2 7

Supplementary Notes 3 7

Supplementary References 8

SUPPLEMENTARY METHODS

In this work, we employed an adapted version of the model developed by Arregui et al.[1] to estimate the long-term impacts of disruptions in TB diagnosis due to the Covid-19 pandemic. The adapted version is fundamentally the same as the original model, except that it introduces a new way of calculating confidence intervals of the outcomes, which works better than the previous one while preserving all the sources of uncertainties.

This model is a deterministic, age-structured model based on ordinary differential equations, where individuals belonging to different age strata are considered to experience different levels of epidemiological risk that translate into age-specific parameter values for at least some of the key dynamical processes. According to this model, the dynamical architecture that defines the disease dynamics within each age group is similar to previous approaches. Other aspects, such as the description of the contact patterns among age groups that lead to pathogen's spreading, or the coupling between populations' aging and transmission dynamics are profusely described in Arregui et al. work[1], where the reader is referred for further details.

In the following lines, we summarise the main aspects of the model, including its Natural History description of the disease, the description of the coupling between the population's aging and disease dynamics, and the propagation of the uncertainty incurred in the outcome. The entire model is described at a much greater level of detail in the original publication[1].

M.tb. transmission dynamics within age strata.

The model is age-structured, including 15 different age groups, 14 of them covering 5 years of age up to 70 years old, and the last one containing all individuals older than 70 years old. Within each age group, we have a class of unexposed individuals –susceptible–, two different latency paths to disease –fast and slow – and six different kinds of disease, depending on its aetiology: -nonpulmonary, pulmonary (smear-positive) and pulmonary (smear-negative)-, and depending on if it is left untreated or treated. After the disease phase, we consider the treatment outcomes contemplated by the WHO data schemes: treatment completion, default, failure, and death[2].

Being the model compartmental, it reproduces the natural history of TB making use of 19 possible reservoirs for individuals, which depends upon the status of the disease. In Supplementary Figure 1 we schematize the whole model.

In a summary, there are several types of possible transitions between the compartments of the TB natural history, which are listed below:

- Infection processes: after contact with an infectious individual, susceptible individuals (S) get infected, entering either the fast (L_F), or slow latency states (L_S).

- Re-infection processes: individuals in the slow latency reservoir can get re-infected, a fraction of which will develop TB fast after re-infection. This is modeled as a transition from L_S to L_F.
- Development of active TB: infected individuals (either L_F or L_S) may develop initially undiagnosed -and thus untreated- TB (D states).
- TB diagnosis: with some delay after the disease onset, TB gets diagnosed and treatment starts (transition from D states to T ones)
- Treatment outcomes: (transitions from T states to R ones) different possible outcomes are possible: –either success or failure/default- (failure of treatment moves individuals to F)
- Disease relapse: (transitions back from T to R)
- Death: active TB patients, either diagnosed or not, are assigned a TB-specific mortality rate.

The specific details about the mathematical parameterization of these and other model transitions are explicitly enumerated and described in the supplementary appendix of Arregui et al. work[1], and the ODE system that governs the evolution in each compartment is also reported in the next section. Altogether, they define the evolution of individuals with time, which can finally get aggregated across age strata to define global estimates of TB burden.

Within this scheme, infections occur after contact between susceptible individuals and infectious ones. If $S(a,t)$ represents the number of susceptible subjects in the age group a, at a given time t, the number of new infections that will be observed will be equal to the product of $S(a,t)$ and the force of infection perceived by that sub-population, $\lambda(a,t)$, which represents the fraction of susceptible individuals who get infected per year. In turn, the force of infection is proportional to the following sum:

$$\sum_{a'} \xi_c(a,a',t)\Upsilon(a',t) \tag{1}$$

where $\Upsilon(a',t)$ is the density of all the infectious individuals within age-group a' at time step t, weighted by their relative infectiousness; and $\xi_c(a,a',t)$ represents the relative contact frequency that an individual of age a has with individuals of age a' at time t, with respect to the overall average of contacts that an individual has per unit time with anyone else. For the computation of the contact matrices used in our model, we have integrated data from different survey studies conducted in African countries (Kenya[3], Zimbabwe[4] and Uganda[5]), to obtain a unique matrix broadly representative of contact structures in Africa[1,6]. Importantly, we also take into account that, as the demographic structure of the population changes, the contact patterns change too[6], being this the origin of the dependence over time we found in matrices.

Ordinary differential equations system

In the previous section, we included the TB natural history which is used in the model as the compartments of the compartmental model. The evolution of the population in each compartment is given by an ordinal differential equation (ODE) which captures the transitions that either introduce new individuals or withdraw them. The following system of differential equations describes, then, the evolution of the different dynamical states of the model:

$$\begin{aligned}\dot{S}(a,t) &= -\lambda(a,t)S(a,t) - ((1-\delta(a-14))S(a,t) - (1-\delta(a))S(a-1,t))/\tau \\ &+ \delta(a)(1-m_c m_d(t))\Delta_N(a,t) + (1-\delta(a))\Delta_N(a,t)S(a,t)/N(a,t)\end{aligned} \tag{2}$$

$$\begin{aligned}\dot{L}_s(a,t) &= (1-p(a))\lambda(a,t)S(a,t) - p(a)q\lambda(a,t)L_s(a,t) - \omega_s L_s(a,t) + \delta(a)m_c m_d(t)(1-p(0))\Delta_N(a,t) \\ &- ((1-\delta(a-14))L_s(a,t) - (1-\delta(a))L_s(a-1,t))/\tau + (1-\delta(a))\Delta_N(a,t)L_s(a,t)/N(a,t)\end{aligned} \tag{3}$$

$$\begin{aligned}\dot{L}_f(a,t) &= p(a)\lambda(a,t)S(a,t) - \omega_f L_f(a,t) + p(a)q\lambda(a,t)(L_s(a,t) + R_{p+N}(a,t) + R_{p-N}(a,t) + R_{npN}(a,t)) \\ &+ p(a)q\lambda(a,t)(R_{p+S}(a,t) + R_{p-S}(a,t) + R_{npS}(a,t) + R_{p+D}(a,t) + R_{p-D}(a,t) + R_{npD}(a,t)) \\ &- ((1-\delta(a-14))L_f(a,t) - (1-\delta(a))L_f(a-1,t))/\tau + \delta(a)m_c m_d(t)p(0)\Delta_N(a,t) \\ &+ (1-\delta(a))\Delta_N(a,t)L_f(a,t)/N(a,t)\end{aligned} \tag{4}$$

$$\begin{aligned}\dot{D}_{p+}(a,t) &= \omega_f \rho_{p+}(a)L_f(a,t) + \omega_s \rho_{p+}(a)L_s(a,t) - \mu_{p+}D_{p+}(a,t) - d(t)D_{p+}(a,t) \\ &- \nu D_{p+}(a,t) + r_N R_{p+N}(a,t) + r_S R_{p+S}(a,t) + r_D R_{p+D}(a,t) + \theta D_{p-}(a,t) \\ &- ((1-\delta(a-14))D_{p+}(a,t) - (1-\delta(a))D_{p+}(a-1,t))/\tau + (1-\delta(a))\Delta_N(a,t)D_{p+}(a,t)/N(a,t)\end{aligned} \tag{5}$$

$$\begin{aligned}\dot{D}_{p-}(a,t) &= \omega_f(1-\rho_{p+}(a)-\rho_{np}(a))L_f(a,t) + \omega_s(1-\rho_{p+}(a)-\rho_{np}(a))L_s(a,t) - \mu_{p-}D_{p-}(a,t) \\ &- \eta d(t)D_{p-}(a,t) - \nu D_{p-}(a,t) + r_N R_{p-N}(a,t) + r_S R_{p-S}(a,t) + r_D R_{p-D}(a,t) - \theta D_{p-}(a,t) \\ &- ((1-\delta(a-14))D_{p-}(a,t) - (1-\delta(a))D_{p-}(a-1,t))/\tau + (1-\delta(a))\Delta_N(a,t)D_{p-}(a,t)/N(a,t)\end{aligned} \tag{6}$$

$$\begin{aligned}\dot{D}_{np}(a,t) &= \omega_f \rho_{np}(a)L_f(a,t) + \omega_s \rho_{np}(a)L_s(a,t) - \mu_{np}D_{np}(a,t) - \eta d(t)D_{np}(a,t) \\ &- \nu D_{np}(a,t) + r_N R_{npN}(a,t) + r_S R_{npS}(a,t) + r_D R_{npD}(a,t) \\ &- ((1-\delta(a-14))D_{np}(a,t) - (1-\delta(a))D_{np}(a-1,t))/\tau + (1-\delta(a))\Delta_N(a,t)D_{np}(a,t)/N(a,t)\end{aligned} \tag{7}$$

$$\begin{aligned}\dot{T}_{p+}(a,t) &= d(t)D_{p+}(a,t) - \Psi T_{p+}(a,t) + \theta T_{p-}(a,t) \\ &- ((1-\delta(a-14))T_{p+}(a,t) - (1-\delta(a))T_{p+}(a-1,t))/\tau + (1-\delta(a))\Delta_N(a,t)T_{p+}(a,t)/N(a,t)\end{aligned} \tag{8}$$

$$\begin{aligned}\dot{T}_{p-}(a,t) &= \eta d(t)D_{p-}(a,t) - \Psi T_{p-}(a,t) - \theta T_{p-}(a,t) \\ &- ((1-\delta(a-14))T_{p-}(a,t) - (1-\delta(a))T_{p-}(a-1,t))/\tau + (1-\delta(a))\Delta_N(a,t)T_{p-}(a,t)/N(a,t)\end{aligned} \tag{9}$$

$$\begin{aligned}\dot{T}_{np}(a,t) &= \eta d(t)D_{np}(a,t) - \Psi T_{np}(a,t) \\ &- ((1-\delta(a-14))T_{np}(a,t) - (1-\delta(a))T_{np}(a-1,t))/\tau + (1-\delta(a))\Delta_N(a,t)T_{np}(a,t)/N(a,t)\end{aligned} \tag{10}$$

$$\begin{aligned}\dot{F}(a,t) &= \Psi f_F^{p+}T_{p+}(a,t) + \Psi f_F^{p-}(T_{p-}(a,t) + T_{np}(a,t)) - \mu_{p+}F(a,t) \\ &- ((1-\delta(a-14))F(a,t) - (1-\delta(a))F(a-1,t))/\tau + (1-\delta(a))\Delta_N(a,t)F(a,t)/N(a,t)\end{aligned} \tag{11}$$

$$\begin{aligned}\dot{R}_{p+N}(a,t) &= \nu D_{p+}(a,t) - r_N R_{p+N}(a,t) - p(a)q\lambda(a,t)R_{p+N}(a,t) \\ &- ((1-\delta(a-14))R_{p+N}(a,t) - (1-\delta(a))R_{p+N}(a-1,t))/\tau + (1-\delta(a))\Delta_N(a,t)R_{p+N}(a,t)/N(a,t)\end{aligned} \tag{12}$$

$$\dot{R}_{p-N}(a,t) = \nu D_{p-}(a,t) - r_N R_{p-N}(a,t) - p(a)q\lambda(a,t)R_{p-N}(a,t) \\ - ((1-\delta(a-14))R_{p-N}(a,t) - (1-\delta(a))R_{p-N}(a-1,t))/\tau + (1-\delta(a))\Delta_N(a,t)R_{p-N}(a,t)/N(a,t) \quad (13)$$

$$\dot{R}_{npN}(a,t) = \nu D_{np}(a,t) - r_N R_{npN}(a,t) - p(a)q\lambda(a,t)R_{npN}(a,t) \\ - ((1-\delta(a-14))R_{npN}(a,t) - (1-\delta(a))R_{npN}(a-1,t))/\tau + (1-\delta(a))\Delta_N(a,t)R_{npN}(a,t)/N(a,t) \quad (14)$$

$$\dot{R}_{p+S}(a,t) = \Psi(1 - f_D^{p+} - f_F^{p+} - f_\mu^{p+})T_{p+}(a,t) - r_S R_{p+S}(a,t) - p(a)q\lambda(a,t)R_{p+S}(a,t) \\ - ((1-\delta(a-14))R_{p+S}(a,t) - (1-\delta(a))R_{p+S}(a-1,t))/\tau + (1-\delta(a))\Delta_N(a,t)R_{p+S}(a,t)/N(a,t) \quad (15)$$

$$\dot{R}_{p-S}(a,t) = \Psi(1 - f_D^{p-} - f_F^{p-} - f_\mu^{p-})T_{p-}(a,t) - r_S R_{p-S}(a,t) - p(a)q\lambda(a,t)R_{p-S}(a,t) \\ - ((1-\delta(a-14))R_{p-S}(a,t) - (1-\delta(a))R_{p-S}(a-1,t))/\tau + (1-\delta(a))\Delta_N(a,t)R_{p-S}(a,t)/N(a,t) \quad (16)$$

$$\dot{R}_{npS}(a,t) = \Psi(1 - f_D^{p-} - f_F^{p-} - f_\mu^{p-})T_{np}(a,t) - r_S R_{npS}(a,t) - p(a)q\lambda(a,t)R_{npS}(a,t) \\ - ((1-\delta(a-14))R_{npS}(a,t) - (1-\delta(a))R_{npS}(a-1,t))/\tau + (1-\delta(a))\Delta_N(a,t)R_{npS}(a,t)/N(a,t) \quad (17)$$

$$\dot{R}_{p+D}(a,t) = \Psi f_D^{p+} T_{p+}(a,t) - r_D R_{p+D}(a,t) - p(a)q\lambda(a,t)R_{p+D}(a,t) \\ - ((1-\delta(a-14))R_{p+D}(a,t) - (1-\delta(a))R_{p+D}(a-1,t))/\tau + (1-\delta(a))\Delta_N(a,t)R_{p+D}(a,t)/N(a,t) \quad (18)$$

$$\dot{R}_{p-D}(a,t) = \Psi f_D^{p-} T_{p-}(a,t) - r_D R_{p-D}(a,t) - p(a)q\lambda(a,t)R_{p-D}(a,t) \\ - ((1-\delta(a-14))R_{p-D}(a,t) - (1-\delta(a))R_{p-D}(a-1,t))/\tau + (1-\delta(a))\Delta_N(a,t)R_{p-D}(a,t)/N(a,t) \quad (19)$$

$$\dot{R}_{npD}(a,t) = \Psi f_D^{p-} T_{np}(a,t) - r_D R_{npD}(a,t) - p(a)q\lambda(a,t)R_{npD}(a,t) \\ - ((1-\delta(a-14))R_{npD}(a,t) - (1-\delta(a))R_{npD}(a-1,t))/\tau + (1-\delta(a))\Delta_N(a,t)R_{npD}(a,t)/N(a,t) \quad (20)$$

where $\delta(a)$ stands for the Dirac delta function ($\delta(x = 0) = 1$ and $\delta(x \neq 0) = 0$). There are three quantities that depend on time: the force of infection $\lambda(a,t)$, the diagnosis rate $d(t)$ and the correction terms $\Delta_N(a,t)$, standing for any demographic variation in the population due to causes foreign to TB and aging.

For further information about the ODE system, and the set of parameters that appear in them, the reader is referred to the supplementary materials of the original source[1], in which they are described exhaustively. In summary, the parameters appearing here represent either probabilities (for example, probability of developing one kind of TB or another, or success probability of under-treatment individuals) or rates (for instance, the rate of progression from slow and fast latency towards developing active TB) that are reported in the literature and capture the progression of individuals across the states of the model.

Population dynamics across age strata: individuals' ageing and demographic evolution.

This model includes the simultaneous description of the disease dynamics across all age groups in an entire population, using parameters that are, in general, dependent on age. Therefore, it is not enough to describe how the sub-populations associated with the disease states evolve, but the model also includes an aging dynamics whereby individuals transit across the different age strata as they get older. In Supplementary Figure 2 we schematize this phenomenon as the transitions between age strata inside each pyramid and the change in the shape of the demographic pyramid with time.

Two additional ingredients that are key to describing the evolution of the population and the aging are then included in our model. First, we consider empirical data and forecasts to model past and future fertility levels, respectively. Second, we introduce continuous correction terms $\Delta_N(a,t)$ that are added or subtracted from the population within the age stratum a at time t while the simulation unfolds.

These terms are calculated dynamically to make the time evolution of the demographic pyramid match the demographic forecasts reported in the United Nations population division database until the end of the simulation. This way, the correction terms $\Delta_N(a,t)$ are distributed among all disease states proportionally to their relative size. These terms represent changes in the population of each stratum that are unrelated to the dynamics of the disease (TB unrelated mortality and migratory fluxes).

In a summary, demographic pyramids in Supplementary Figure 2 evolve with time in such a way that individuals transit inside the pyramid as they age, but also we update the whole population in a data-driven way. That is, we update the number of individuals inside each age strata according to the prospects of the UN population division, where the correction terms $\Delta_N(a,t)$ are used for this purpose. This assures that both the aging and the demographic prospects are included in the model, capturing the evolution of the population.

Propagation of the uncertainty

The uncertainty sources[1] are summarized here:

- Parameters associated with the Natural History of the disease are treated as totally independent uncertainty sources.
- TB burden estimations provided by the WHO. Based upon several case notifications surveilled in each country, the World Health Organization provides estimations for incidence and mortality rates for almost all countries.
- Demographic structures that are reported in the form of demographic pyramids with 15 age groups, the first 14 from 0y to 70y each 5y, and the last one comprises 70+y. Each group has its uncertainty but we modify them as a whole and not as 15 independent uncertainty sources.
- Contact matrices, whose uncertainty comes from the variability between studies used for building regional contact matrices.

Those uncertainty sources are managed independently and their contributions to the uncertainty of a certain model outcome x are to be evaluated. On the original version of the model[1] the uncertainty of an outcome was translated by doing a sensibility analysis that accounts for the variation produced in the outcome when the median value of an uncertainty source is changed by its extreme values and the uncertainty is built by grouping individual sensitivities according to the type of input data for building credibility intervals and significance levels for the outcome.

In this work, thus, this procedure is deprecated and has changed radically, as the old procedure was conservative in the sense that for each uncertainty source the worst-case scenario was assumed *always*, which is not always a realistic approach to spreading. This approach is replaced, and a probabilistic procedure is implemented. The process performed in the new version of the model is described here:

1. Generate a set of **N** new values of the uncertainty sources taking into account the original value and its CI. The generation of new values is not trivial as we cannot treat all the uncertainty sources in the same way. Further details of the generation procedure are explained in the next section.
2. Calibrate the model for each new set of parameters.
3. Run the model for each new set of parameters, thus obtaining a set of N values for a certain outcome.
4. The outcome is obtained as the median value and the CI interval as the 95% quantile of the set of outcomes.

Proceeding this way we can generate a CI for any outcome the model can give, assuring that all the uncertainty sources are taken into account. The generation process of the new values for each uncertainty source is described in the following section.

Generation of new values for the uncertainty sources

In general, we assume that the reported confidence intervals of each value can be interpreted as coming from a normal distribution, thus being the reported value the mean and building the standard deviation from the CI, so we have $p_i \sim \mathcal{N}(\mu,\sigma)$.

This allows us to generate random numbers under those normal distributions that serve as our new value for the uncertainty source, but caution is needed, as there are several points we must consider. First, some parameters that belong to the interval $\in [0,\infty)$, so their normal distribution must be renormalized in such a way that all the probability lies within the $[0,\infty)$ interval. Other parameters represent probabilities, so not only do they lie in the interval $[0,1]$ but also are bounded to some other parameters. As an example, if we take the treatment success probability and calculate a new value that is higher than the original, then the probability of not succeeding must decrease accordingly.

Here, the random number generation procedure trust in R, as we use the random number generators included in the base package (R 3.4.4).

In the case of contact matrices, we consider each value of the matrix as independent from the others, as each number is obtained through surveys performed in all age groups, and represents the contact they establish with the other age groups. This means that the matrices are not symmetrical in the sense that they do not report the same number of contacts between age group **a** towards age group **a'** as contacts from **a'** to **a**, so they do not show this constraint. Thus, a new matrix is obtained by taking each $M_{a,a'}$ and recalculating it within its distribution. As the number of contacts or the contact rate must lie within the interval $\in [0, \infty)$, they cannot be negative.

Moving to the TB burden, there are two magnitudes reported here, incidence and mortality per million population from the year 2000 to the year 2018. As our model calibrates both the infection force and the diagnosis rate based on this data, we can modify incidence and mortality separately. Nevertheless, values inside each block must be changed in the same way, so if the new incidence in the year 2000 is higher than the original, then all the incidence values of the other years must be higher than their previous values too, as we are moving towards a high incidence setting. Then, we generate new values for the TB burden by drawing a Z-score from the normal distribution $\mathcal{N}(0,\, 1)$ and then using it for calculating the new incidence of each year j, thus:

$$i^j_{\text{new}} = i^j_{\text{orig}} + Z \cdot \sigma^j \tag{21}$$

where σ^j is the standard deviation of the normal distribution of the j-th year. Then this is repeated with a new Z-score for the mortality so both incidence and mortality are modified as a block.

Finally, in dealing with demography we decided to act on the shape of the demographic pyramid instead of modifying the global population. This way the change from one year to another is not too abrupt. Let's denote an arbitrary age group as a so $a \in [1, 15]$. A new Z-score, namely Z', is drawn from $\mathcal{N}(0,\, 1)$ and a new value for the base of the pyramid (a=1) is calculated by:

$$n(a = 1)_{\text{new}} = n(a = 1)_{\text{orig}} + Z \cdot \sigma(a = 1) \tag{22}$$

Then, we modify the rest of the age groups from the base to the top of the pyramid but using a Z-score given by:

$$Z(a, Z') = M(Z') \cdot a + K(Z') \tag{23}$$

where $M(Z') = -\frac{1}{7}Z'$ and $K(Z') = \frac{8}{7}Z'$. This assures that $Z(1) = Z'$ and $Z(15) = -Z'$, which implies that the excess or lack of population in the base groups balance out by the contrary effect in the top of the pyramid, preserving the total population. Consequently, the new value for any group is given by:

$$n(a)_{\text{new}} = n(a)_{\text{orig}} + Z(a, Z') \cdot \sigma(a) \tag{24}$$

Up to this point, we have been able to generate a new set of values for all the uncertainty sources described before. Finally, for each one of these sets, the model needs to be calibrated.

SUPPLEMENTARY NOTES 1

Mobility patterns, Covid-19 influence, and modeling decisions: In this study, we opt to introduce the pandemic disruption as a multiplicative factor that either increases or decreases the expected diagnosis value if the pandemic had never happened and evaluate the changes that occur in the forecast. This multiplicative factor, based on WHO and local reports, accounts not only for the lockdowns but for the whole period in which the healthcare system is disrupted, which is, generally, longer than the lockdown itself.

As facemasks and social distancing have been globally introduced as a countermeasure for avoiding Covid-19 transmission, some effect is expected over the transmission of other airborne diseases such as TB. Interestingly enough, if we observe the changes in mobility according to Google's data[7], along with the Covid-19 confirmed cases and the reduction in the TB notifications in India from Feb 2020 to Oct 2021[8] (see Supplementary Figure 3), we realize that during high-Covid-19 burden periods, where more strict measures are implemented, presence in households and Grocery & pharmacy places increases. Moreover, changes in mobility patterns suggest that, during several phases of the pandemic, measures forced individuals to interact in closed spaces.

From Supplementary Figure 3 we also learn that those high-incidence Covid-19 periods, and when mobility changes drastically, a match with periods of low TB notification when compared to the same period in 2019 is found. This suggests that neither arguing about rises nor decreases in transmission seems to have enough support from data.

SUPPLEMENTARY NOTES 2

Alternative scenario by force of infection and treatment completions: In the model, the force of infection is calculated as:

$$\lambda(a,t) = \beta(t)\sum_{a'} M(a,a',t)\Upsilon(a',t) \tag{25}$$

where $\beta(t)$ is a half sigmoid calibrated by the model, $M(a,a',t)$ represents the relative contact frequency that an individual of age a has with individuals of age a' at time t, with respect to the overall average of contacts that an individual has per unit time with anyone else. $\Upsilon(a',t)$, on the other hand, represents the weighted density of all the infectious individuals within age-group a' at time step t. All together build the force of infection, which represents the rate at which infection occurs at time step t for a susceptible individual in age-group a.

Aiming to characterize the effect of a possible modification in $\lambda(a,t)$, we perform a sensitivity analysis over the TB burden forecasts of the model in the four countries of the study. Given the information presented in Supplementary Figure 3, it is difficult to quantify a variation in the force of infection, as mobility changes suggest that individuals left public spaces and frequented more private and closed spaces. At the same time, the non-pharmaceutical interventions implemented to halt COVID-19 transmission might have had an effect also on the transmission of TB. Early evidence shows that this is not the case and that the transmission of TB has remained constant or even has increased in certain contexts[9,10].

Thus, we perform our sensitivity analysis introducing a multiplicative factor that comprises both increases and decreases evenly distributed around the base value in the range $\beta_r \in [0.85, 1.15]$, representing variations from -15% to 15% of the base value. The new value of the force of infection is thus obtained as:

$$\bar{\lambda}(a,t) = \beta_r \lambda(a,t) \tag{26}$$

Regarding the treatment completion, in the main text a fixed value of $\eta = 0.788$ multiplicative factor is adopted, as literature findings support it. Here, we also decided to explore a more conservative case in which the multiplicative factor is raised to $\eta = 0.90$, which allows more patients to be treated quickly in the model. Then, we perform our simulations and produce forecasts for every alternative scenario and present them in two panels, one for each value of the treatment completion. Supplementary Figure 4 shows the different forecasts when the force of infection is modified accordingly to the multiplicative factor β_r for the same value as in the main text, while Supplementary Figure 5 presents the forecasts with the alternative treatment completion value.

In Supplementary Figure 4 the results for the sensitivity over $\lambda(a,t)$ with a treatment completion scale factor of 0.788, and the results are, as expected, highly dependent upon the force of infection values. That's because the infections in the model are mainly driven through primary infections, i.e., those that occur upon susceptible individuals. Primary infections are highly dependent on $\lambda(a,t)$ too, as in the model they are calculated as the sum over all age groups a of the product $\lambda(a,t)S(a,t)$, thus, the fraction of susceptible individuals of age a that gets infected in time step t. Nevertheless, $\lambda(a,t)$ also affects reinfections, which ultimately leads to a high reduction of the expected burden if it decreases, along with an increment of incidence and mortality if it decreases.

Moreover, in Supplementary Figure 5 the results for the sensitivity over $\lambda(a,t)$ with a treatment completion scale factor of 0.90 are shown. We recover the same qualitative behavior that in the previous case, but not quantitatively, as all future burdens are lower because we are allowing more sick individuals to be treated than in the previous scenario during the pandemic period.

SUPPLEMENTARY NOTES 3

Recurrent bumps: Alternative scenario where the disruption lasts longer than expected: Given the current situation with COVID-19 variants spreading, it is not clear if the pandemic is reaching an end soon. Under those circumstances, we decided to perform a sensitivity analysis in which we explore the hypothetical scenario of having an extra disruption in diagnosis similar to the one reported in the data.

Thus, we perform a new set of simulations in which we forecast both incidence and mortality when another bump equal to the fitted ones is introduced right after the end of the former. In Indonesia, Kenya, and Pakistan, this is trivial, as there is only one big bump in diagnosis reported, whereas, in India, there were two separate bumps, a first, big one, and a second, narrow one. In this situation, we analyzed separately each case, studying the case of repeating either the first bump or the narrowest, second bump. The results of this analysis are reported in Supplementary Figure 6, where diagnosis rates, incidence temporal series, and mortality in 2035 are reported in each scenario.

We observed that, if another disruption is to happen, an even greater increase in mortality occurs in every country, which leads to more deaths associated with the diagnosis disruptions. Moreover, incidence levels, especially in Indonesia, but happening in all countries, need more time to recover the levels of the baseline scenario, risking, even more, the END TB goals.

SUPPLEMENTARY REFERENCES

[1] S. Arregui, M. J. Iglesias, S. Samper, D. Marinova, C. Martin, J. Sanz, and Y. Moreno, Proceedings of the National Academy of Sciences **115**, E3238 (2018), https://www.pnas.org/content/115/14/E3238.full.pdf.
[2] W. H. Organization, Global tuberculosis report 2021 (2021).
[3] M. C. Kiti, T. M. Kinyanjui, D. C. Koech, P. K. Munywoki, G. F. Medley, and D. J. Nokes, PloS one **9**, e104786 (2014).
[4] A. Melegaro, E. Del Fava, P. Poletti, S. Merler, C. Nyamukapa, J. Williams, S. Gregson, and P. Manfredi, PloS one **12**, e0170459 (2017).
[5] O. l. P. de Waroux, S. Cohuet, D. Ndazima, A. Kucharski, A. Juan-Giner, S. Flasche, E. Tumwesigye, R. Arinaitwe, J. Mwanga-Amumpaire, Y. Boum, *et al.*, BMC infectious diseases **18**, 1 (2018).
[6] S. Arregui, A. Aleta, J. Sanz, and Y. Moreno, PLoS computational biology **14**, e1006638 (2018).
[7] Google, Covid-19 community mobility reports (2021).
[8] G. of India, Nikshay online tool for monitoring tb control programme (2021).
[9] M. Aznar, J. Espinosa-Pereiro, N. Saborit, N. Jové, F. S. Martinez, S. Pérez-Recio, A. Vitoria, S. Sanjoaquin, E. Gallardo, J. Llenas-García, *et al.*, International Journal of Infectious Diseases (2021).
[10] Y. Geng, G. Li, and L. Zhang, Frontiers in Public Health **9** (2021).

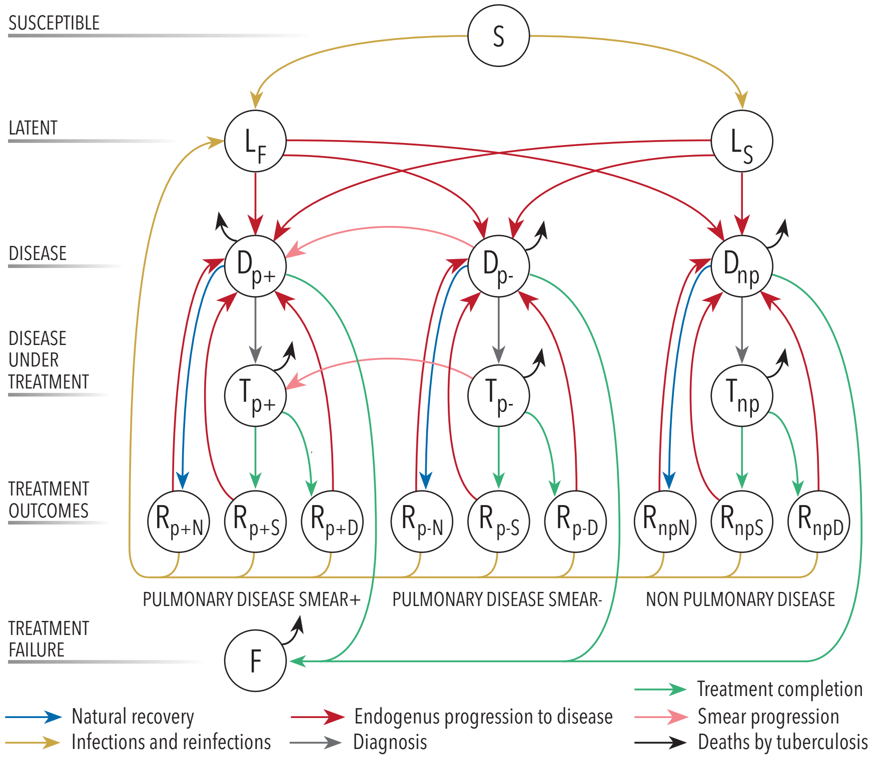

Supplementary Figure 1. Natural history of the disease employed in the mathematical model[1]. Individuals are classified according to their epidemiological status, which can be: S: susceptible. L: latent. D: (untreated) disease, T (treated) disease, R recovered, F: failed recovery . The types of TB considered here are: p+: Pulmonary Smear-Positive, p-: Pulmonary Smear-Negative, and np: Non-pulmonary. Finally, individuals under treatment end it for three different sub-types of recovery, which are: N: Natural, S: Successful, and D: Default (abandon of treatment).

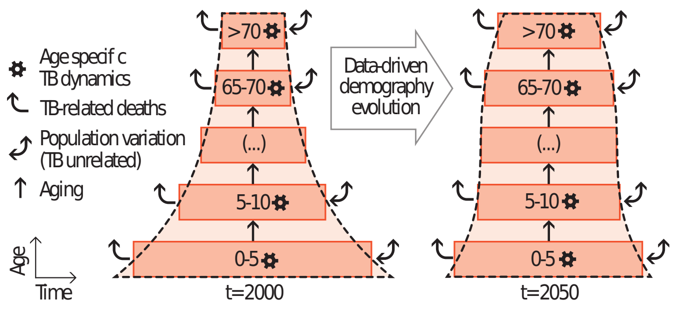

Supplementary Figure 2. Scheme of the coupling between TB dynamics and demographic evolution. The transmission model summarized in A describes the evolution of the disease in each age group, including the removal of individuals due to TB mortality (curved arrows). The evolution of the total volume of each age stratum is corrected (bidirectional arrows: TB-unrelated population variations) to make the demographic pyramid evolve according to UN prospects.

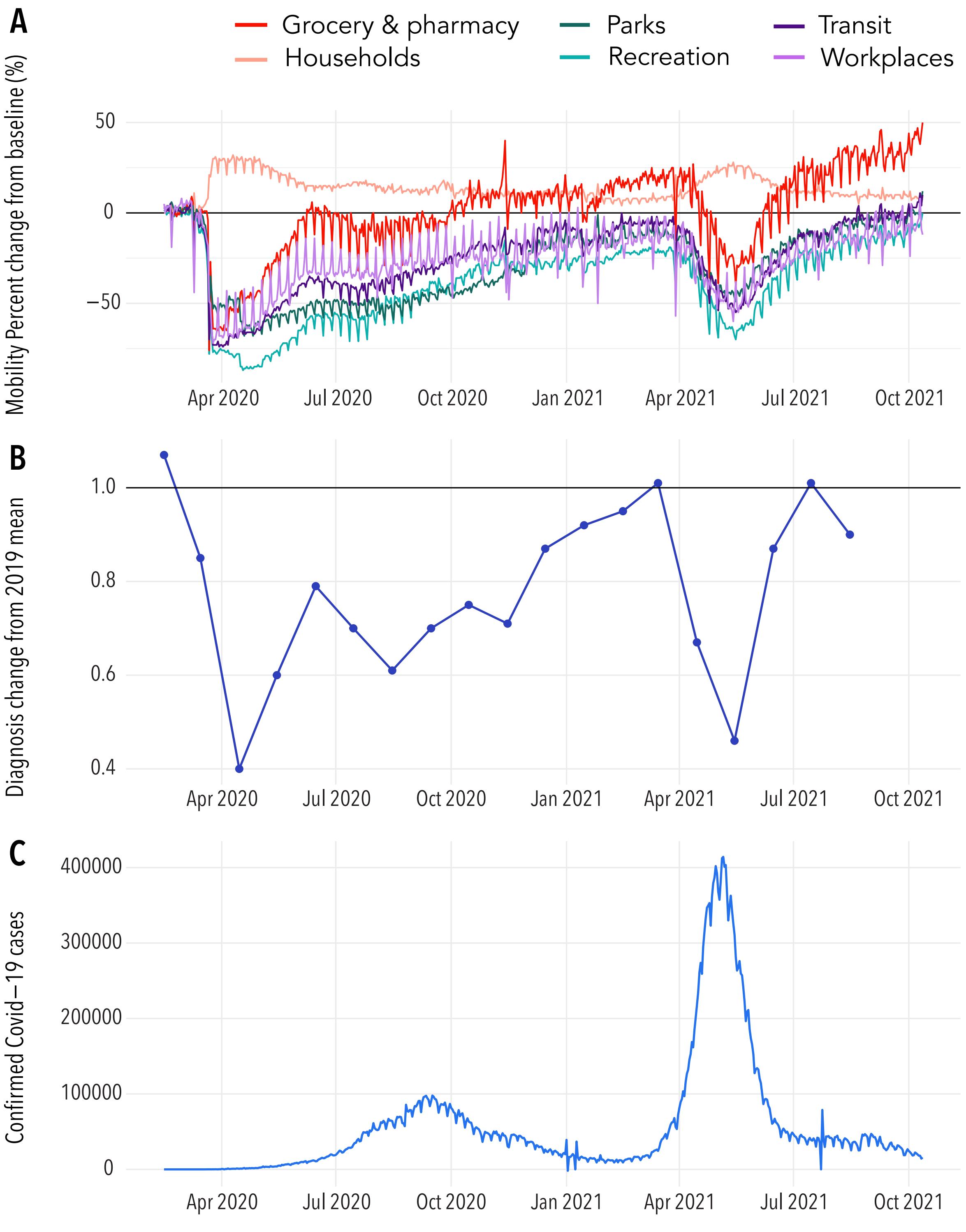

Supplementary Figure 3. Comparison between the changes in mobility, the changes in TB notification cases, and the Covid-19 confirmed cases for the same period in India. A: Percent change in mobility in selected environments when compared to the same period in 2019. B: Per unit change in the TB notifications across public and private hospitals in India, according to Nikshay's data[8]. C: Covid-19 Confirmed cases in India.

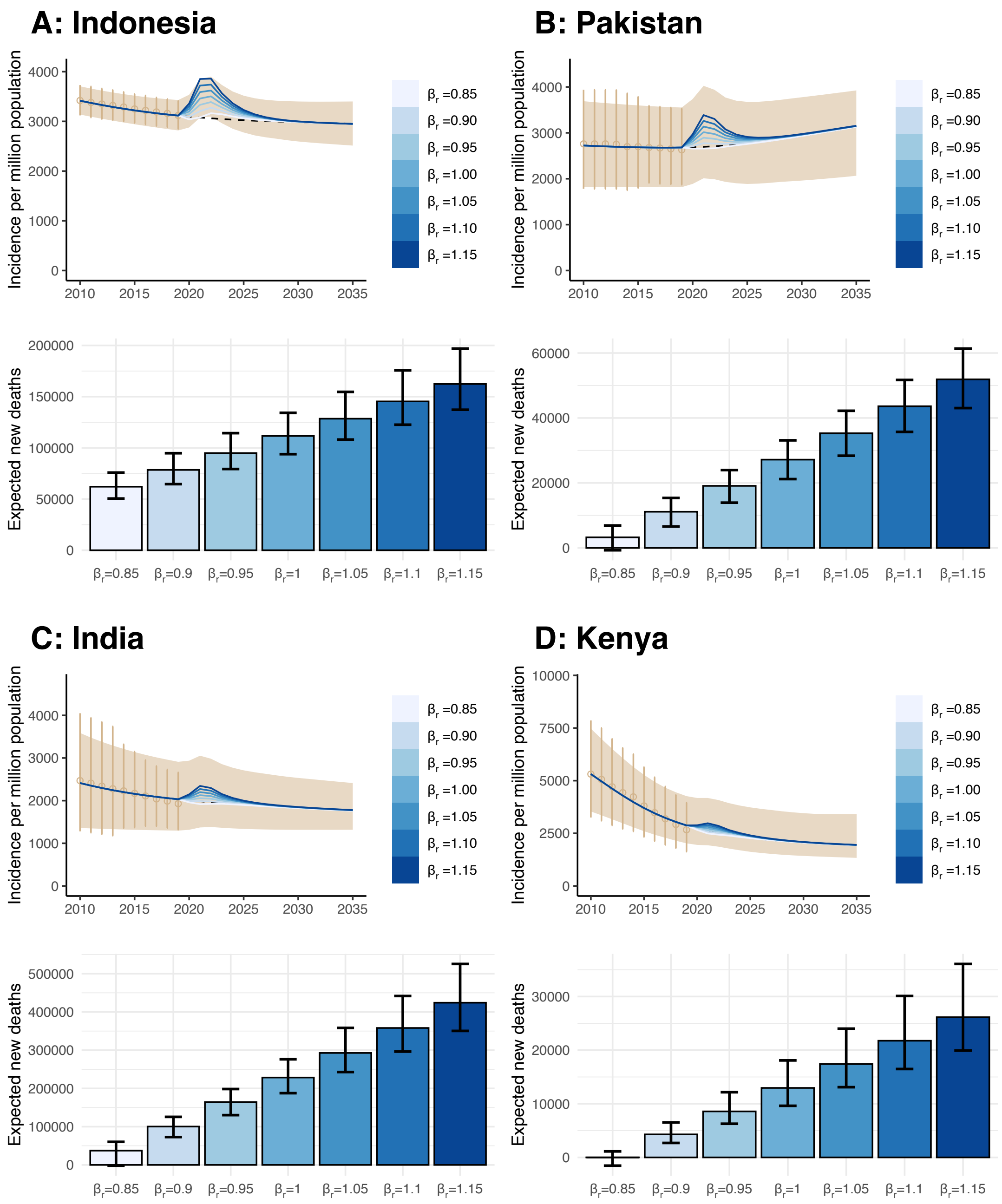

Supplementary Figure 4. TB burden under alternative scenarios in selected countries for a fixed value of $\eta = 0.788$. In each country, the top figure shows the baseline changes in incidence per million population when β_r is introduced. The peak area changes with the value of β_r, either increasing ($\beta_r > 1$) or decreasing ($\beta_r < 1$) from the $\beta_r = 1$ standard scenario. The bottom figure shows the expected additional deaths measured in 2035 (thus, the integral of the peak) when compared to the forecast without Covid-19. Colors for each value of β_r are the same in both figures.

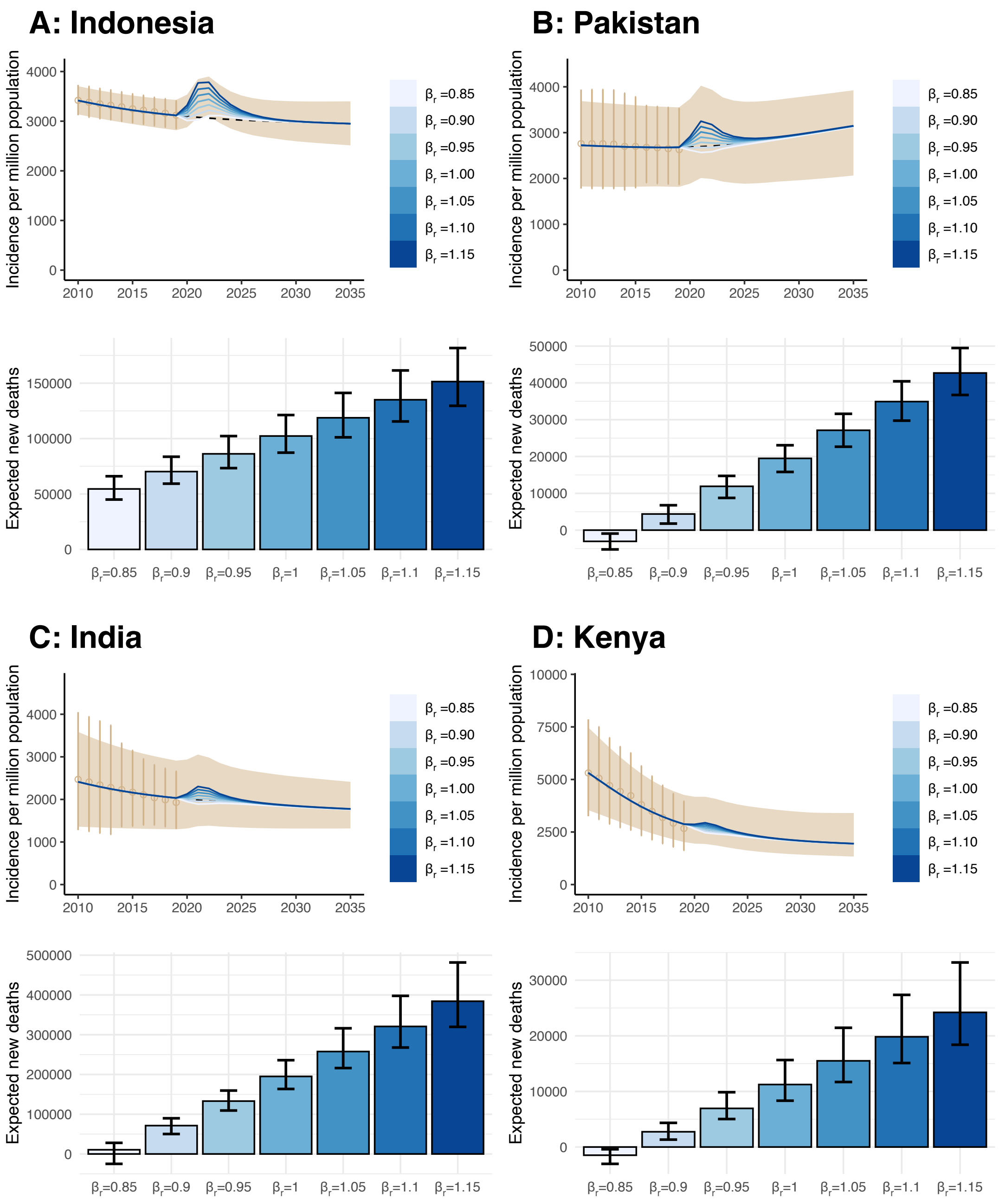

Supplementary Figure 5. TB burden under alternative scenarios in selected countries for a fixed value of $\eta = 0.9$. In each country, the top figure shows the baseline changes in incidence per million population when β_r is introduced. The peak area changes with the value of β_r, either increasing ($\beta_r > 1$) or decreasing ($\beta_r < 1$) from the $\beta_r = 1$ standard scenario. The bottom figure shows the expected additional deaths measured in 2035 (thus, the integral of the peak) when compared to the forecast without Covid-19. Colors for each value of β_r are the same in both figures.

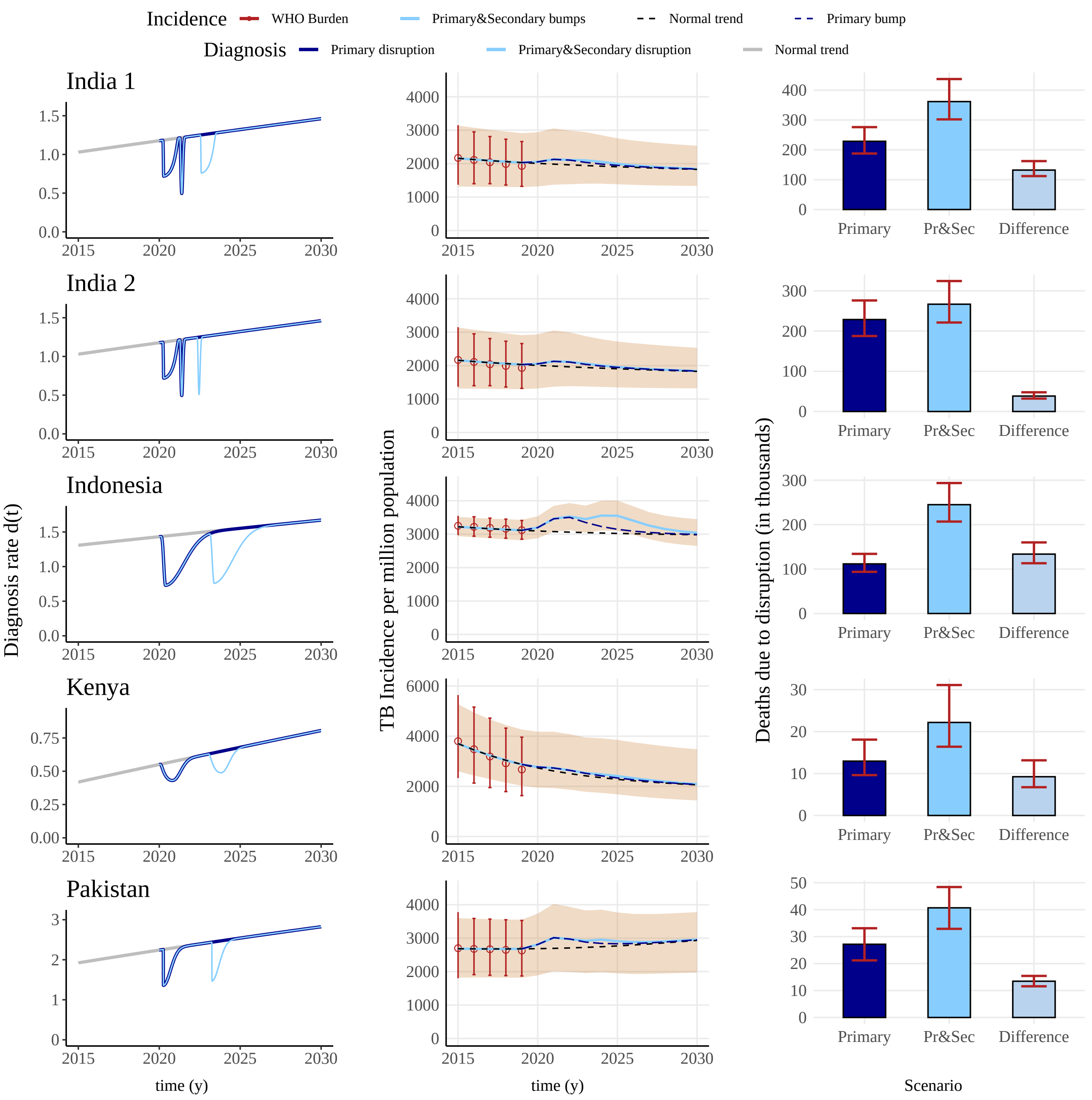

Supplementary Figure 6. Diagnosis and TB burden in alternative scenarios. The first column reports the diagnosis rate $D(t)$, in each country, in the baseline scenario (grey dotted line) compared to the COVID-19 primary disruption and the hypothetical second disruption (blue lines). The second column reports TB incidence temporal series in each country in the same scenarios as before. If a secondary disruption is introduced, baseline incidence levels take more time to be reached. Finally, the third and last column reports the expected mortality in the year 2035 comparing the primary and primary plus secondary scenarios. In the latter, an increase in mortality leads to even more deaths caused by the pandemic disruption of TB care.
