# Supplementary material for: Modeling the impact of COVID-19 on future tuberculosis burden
Source: Commun Med (Lond). 2022 Jun 29;2:77. doi: 10.1038/s43856-022-00145-0 (PMC9243113; doi:10.1038/s43856-022-00145-0)
Supplement: Supplementary file 2 — Description of Additional Supplementary Files [file 43856_2022_145_MOESM2_ESM.pdf]

Description of Additional Supplementary Files

File Name: Supplementary Data 1

Description: Supplementary Data for Figure 1. The first sheet contains the temporal series of diagnosis rates in the pandemic and the previous scenario. The second sheet contains the drops in TB notifications. All series are divided by country.

File Name: Supplementary Data 2

Description: Supplementary Data for Figure 2. Each column represents the incidence temporal series present in figure 2. The first column is the time. The next three are the central incidence in the pandemic scenario and its 95% CI. The last column is the forecasted incidence without the COVID-19 pandemic.

File Name: Supplementary Data 3

Description: Supplementary Data for Figure 3. Each column represents the set of TB deaths across all simulations, per country, and the difference between the COVID-19 scenario and the previous one.

File Name: Supplementary Data 4

Description: Supplementary Data for Figure 4. Each column represents the temporal series of the diagnosis rate in each possible scenario: Without the COVID-19 pandemic, with the COVID-19 pandemic, and with the COVID-19 pandemic plus an intervention that boosts diagnosis at the end of the pandemic.

File Name: Supplementary Data 5

Description: Supplementary Data for Figure 5. The first two columns are the combination of parameters that boost the diagnosis rate. The last column is the Mortality change (%) between the scenario with the COVID-19 pandemic and the same scenario plus the intervention.

File Name: Supplementary Data 6

Description: Supplementary Data for Supplementary Figure 3. Each sheet contains the date below each one of the plots in Supplementary Figure 3. The first one contains the changes in mobility as a temporal series, the second one contains the TB notifications during the pandemic and normalized by 2019 mean, and the last one contains the confirmed COVID-19 cases in the same period.

File Name: Supplementary Data 7

Description: Supplementary Data for Supplementary Figure 4 & 5. The first four sheets contain the temporal series of incidence in each one of the alternative scenarios studied, per country. In the column names, that are of the form Inc_XXX_YYY, XXX represents the reduction in treatment availability (90 for a 10% reduction and 78 for a 22% reduction), whereas YYY represents the value that modifies transmission (095 for a 5% reduction, 105 for a 5% rise,…)

The last sheet contains, per country, the TB mortality associated with each one of the previous scenarios.

File Name: Supplementary Data 8

Description: Supplementary Data for Supplementary Figure 6. The first two sheets contain the data of the diagnosis rate in the primary bump scenario and the primary plus secondary bumps scenario, per country, and as a temporal series. The following two sheets contain the TB incidence and mortality data in each scenario as a temporal series and per country, and the last one contains the aggregated mortality in the set of simulations in each scenario and the difference between scenarios.
